# Arousal from hibernation increases blood oxygen saturation in 13-lined ground squirrels

**DOI:** 10.1242/jeb.249830

**Published:** 2025-04-28

**Authors:** Brynne M. Duffy, Catherine M. Ivy, James F. Staples

**Affiliations:** ^1^ Department of Biology, The University of Western Ontario, London, ON, Canada, N6A 3K7; ^2^ Department of Biology, The University of Saskatchewan, Saskatoon, SK, Canada, S7N 5E2

**Keywords:** Hypoxia, Re-oxygenation, Torpor, Interbout euthermia, *S*a_O_2__, *Ictidomys tridecemlineatus*

## Abstract

Hibernating *Ictidomys tridecemlineatus*, 13-lined ground squirrels, are considered models of ischaemia–reperfusion tolerance, as both tissues and isolated mitochondria withstand anoxia followed by rapid re-oxygenation *in vitro*. This tolerance is likely adaptive, protecting against damage during the numerous arousals from torpor throughout the hibernation season. O_2_ availability is likely low during torpor, but suppressed metabolism lowers O_2_ demand, potentially mitigating hypoxic stress. During arousal to interbout euthermia (IBE), heart rate, blood pressure and ventilation increase rapidly, suggesting increased O_2_ availability, but tissue oxygenation has not been measured during arousal or IBE in 13-lined ground squirrels. Using pulse-oximetry collars, we characterized dramatic increases in O_2_ availability during arousal; carotid artery O_2_ saturation rose from as low as 35% early in arousal to 87% during IBE. These changes closely followed rising heart rate. Our results demonstrate that hibernating 13-lined ground squirrels survive profound O_2_ deprivation early in arousal and rapid O_2_ influx as arousal progresses.

## Teaser

**Highlighted Article:** Evidence that during arousal from hibernation, 13-lined ground squirrels undergo rapid, 2.5-fold increases in arterial oxygen saturation, suggesting transient hypoxia–reoxygenation.

## INTRODUCTION

**Fig. 1.**
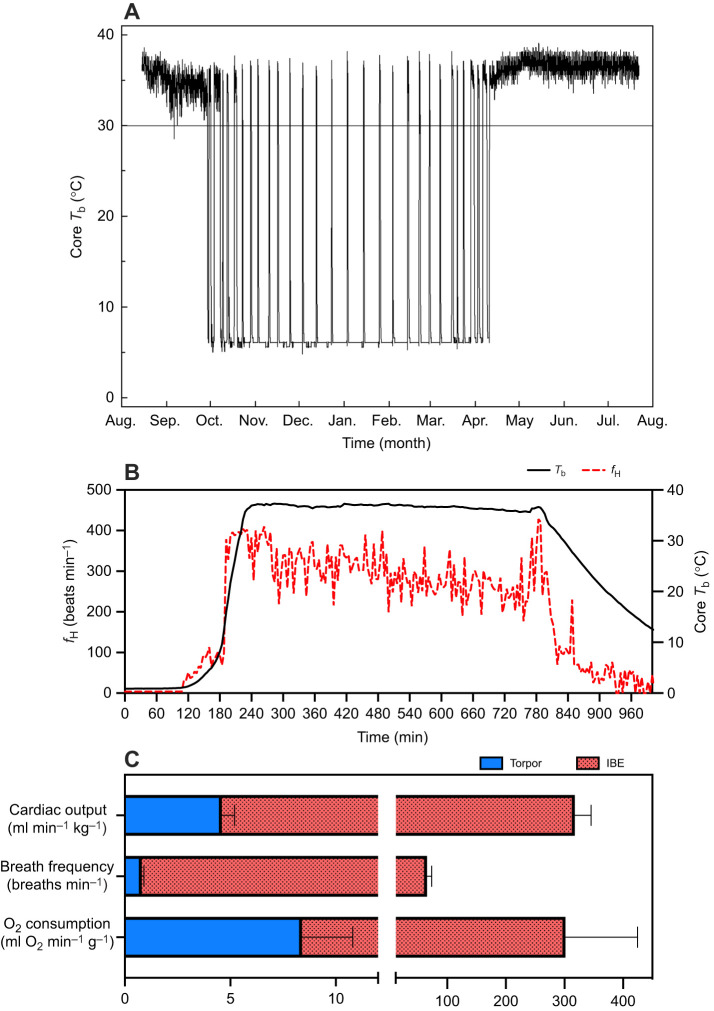
**Physiological metrics that change throughout hibernation in 13-lined ground squirrels (*Ictidomys tridecemlineatus*).** (A) Body temperature (*T*_b_) in a 13-lined ground squirrel during hibernation. (B) Heart rate (*f*_H_; red dashed line) and *T*_b_ (solid black line) fluctuations during an arousal; adapted from MacCannell et al. (2018), with permission. (C) Comparison of cardiac output (from Popovic, 1964), breathing frequency (from Sprenger and Milsom, 2022) and whole-animal oxygen consumption (from Muleme et al., 2006) between torpor and interbout euthermia (IBE). Data are means±s.e.m.

Hibernators evade winter's energetic demands through metabolic suppression. In ground squirrels, hibernation involves cycling between torpor and interbout euthermia (IBE). 13-Lined ground squirrels (*Ictidomys tridecemlineatus*) can experience up to 20 torpor bouts per season, lasting 6–15 days with 90% metabolic suppression and body temperatures (*T*_b_) of 4–6°C (Muleme et al., 2006; Fig. 1A). Torpor is interrupted by arousals to IBE, where metabolism and *T*_b_ return to high values (similar to summer values) for 6–10 h before animals re-enter torpor (Fig. 1A). Low *T*_b_ alone typically induces a leftward shift in the haemoglobin–oxygen (Hb–O_2_) equilibrium curve, as *P*_50_, the partial pressure of O_2_ at which haemoglobin is 50% saturated, decreases with temperature. During torpor, *P*_50_ decreases in hibernating ground squirrels, corresponding with suppressed metabolism (Revsbech et al., 2013; Maginniss and Milsom, 1994; Revsbech and Fago, 2017). However, early in arousal, metabolic demand rises before *T*_b_, increasing the risk of hypoxia, especially before shivering thermogenesis commences.

Hb concentration and haematocrit (Hct) are also important for O_2_ delivery; data on these values vary. For instance, one study reported declines in red blood cell count, Hb and Hct during torpor compared with summer (Spurrier and Dawe, 1973), but later research found no significant changes (Cooper et al., 2016a). Blood ion and metabolite concentration can help contextualize haematological changes; high blood Na^+^ may indicate dehydration, leading to overestimates of Hb and Hct as a result of decreasing plasma volume. Moreover, decreases in blood glucose may indicate anaerobic metabolism from reduced O_2_ availability (Abu et al., 2018).

During arousal, heart rate (*f*_H_), cardiac output and ventilation increase to accommodate increased metabolic rate (Fig. 1B,C). Blood pressure also increases during arousal, rising from 60/30 mmHg (systolic/diastolic) in torpor to 140/100 mmHg during IBE (Lyman, 1960), and blood flow to the heart of 13-lined ground squirrels increases 8-fold (Bullard and Funkhouser, 1962). During torpor in 13-lined ground squirrels, coagulation factor activity and platelet count decline (Cooper et al., 2016b), and fibrinolysis is 3-fold faster (Bonis et al., 2019), indicating minimal resistance to blood flow early in arousal.

During arousal, a 2-fold increase in both ventilation (Sprenger and Milsom, 2022) and cardiac output (Popovic, 1964) likely correspond with rapid increases in O_2_ delivery, which can increase oxidative damage in other animal models (Granger and Kvietys, 2015). Even 13-lined ground squirrels have higher oxidative damage to lipids and proteins in some tissues during IBE compared with torpor (Duffy and Staples, 2022). 13-Lined ground squirrel arousals are often considered similar to ischaemia–reperfusion (I–R) (Bonis et al., 2019; Otis et al., 2017; Kurtz and Carey, 2006). I–R is characterized by a restriction of tissue blood supply, followed by rapid restoration of blood flow and re-oxygenation, and is often associated with pathological conditions such as ischaemic stroke or myocardial infarction. In humans, an arterial oxygen saturation below 55% indicates ischaemia and severe O_2_ deprivation to tissue (Yu et al., 2020). While ischaemic hypoxia can cause significant injury itself, the damage is exacerbated during the reperfusion event, when sudden increases in O_2_ result in excess reactive oxygen species (ROS) production (Granger and Kvietys, 2015). Elevated ROS production rates can exceed antioxidant capacity, causing oxidative damage to cellular macromolecules, including proteins, lipids and DNA.

Resistance to I–R injury is a characteristic of hibernating mammals. For example, hepatic cells from the facultative hibernator Syrian hamster (*Mesocricetus auratus*) did not show DNA damage following I–R, whereas lab mice (C57BL/6) cells did (Pissas et al., 2025). At the whole-animal level, in the summer phenotype, arctic ground squirrels (*Spermophilus parryii*) survive haemorrhagic shock better than rats (Sprague–Dawley; non-hibernators) (Bogren et al., 2014). Within a species, the hibernation phenotype further enhances ischaemia–reperfusion tolerance. Livers from hibernating 13-lined ground squirrels tolerate cold ischaemia followed by warm reperfusion for longer and with less damage than livers from summer 13-lined ground squirrels or rats (Lindell et al., 2004). This resistance is hypothesized to persist because of acquired adaptations that allow small hibernators to endure dozens of arousals every hibernation season. However, the mechanisms of I–R resistance in hibernators remain unknown.

Despite the documented changes in cardiovascular function during arousal (Fig. 1C), whether tissues become hypoxic in 13-lined ground squirrels remains unclear. In torpid arctic ground squirrels, arterial O_2_ tension (*P*a_O_2__) and arterial Hb O_2_ saturation (*S*a_O_2__) are comparable to those in non-hibernating rats (Sprague–Dawley; Ma et al., 2005), indicating that reductions in O_2_ delivery are matched by reductions in tissue O_2_ demand in torpor. Conversely, lactate accumulates in red blood cells of arctic ground squirrels during torpor, suggesting anaerobic metabolism (Gehrke et al., 2019). Furthermore, in 13-lined ground squirrels, levels of hypoxia-inducible transcription factor-1 (HIF-1α) increase in skeletal muscle, brown adipose tissue and liver during torpor, indicating widespread hypoxia (Maistrovski et al., 2012).

While it has long been predicted that 13-lined ground squirrels experience a significant influx of O_2_ during arousal when *f*_H_ (Fig. 1B), ventilation and blood pressure (Fig. 1C) increase rapidly, other variables, including *T*_b_ and metabolic rate, may confound this effect. We hypothesized that an increased O_2_ demand during arousal is matched by increased O_2_ supply and predicted that O_2_ availability would be low in early arousal but increase as arousal progresses. To test this hypothesis, we measured *S*a_O_2__ during the transition from torpor to IBE using pulse oximetry of blood flow through the carotid arteries. Although changes in Hct and/or Hb could also affect O_2_ delivery during arousal, taking multiple blood samples from arousing animals was not feasible. We did, however, compare these values, as well as some blood ion and glucose concentrations, between 13-lined ground squirrels in deep torpor and in summer.

## MATERIALS AND METHODS

### 13-Lined ground squirrels

All procedures were performed at Western University (London, ON, Canada, 251 m above sea level) following an approved animal use protocol (2020-034), conforming to the Canadian Council on Animal Care guidelines. The 13-lined ground squirrels, *Ictidomys tridecemlineatus* (Mitchill 1821), were live-trapped in Carman, MB, Canada (49°30′N, 96 98°01′W) or bred in captivity, following established husbandry protocols (Vaughan et al., 2006). Husbandry protocols followed the methodology previously outlined in Duffy and Staples (2022). Summer 13-lined ground squirrels were kept at 22±3°C, with a photoperiod adjusted weekly to match the conditions in Carman. Winter 13-lined ground squirrels were transferred to walk-in environmental chambers where the ambient temperature (*T*_a_) was decreased by 1°C per day until it reached 4±2°C, and the photoperiod was set to 24 h of darkness to replicate fossorial (burrow) conditions.

In hibernating animals, torpor bouts were tracked by monitoring the presence of un-shredded paper towels daily (Hutchinson et al., 2024). All squirrels used in this study were adults that had previously undergone at least one hibernation season. We used pulse oximetry to assay arousing 13-lined ground squirrels (three females and six males) in December 2022 and summer 13-lined ground squirrels (two females and two males) in June 2023. We collected blood samples from 17 torpid squirrels (nine females, eight males) in January 2024 and from a different group of 11 summer squirrels (six females, five males) in June 2024, which were also used for heart mass comparisons and separate studies on mitochondrial metabolism.

### Pulse oximetry

**Fig. 2.**
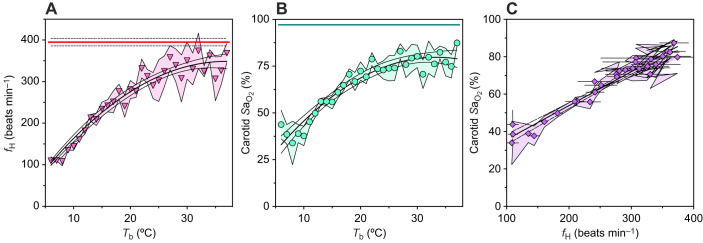
**Increases in *f*_H_ and oxygen saturation during arousal.** (A,B) Increase in *f*_H_ (A) and carotid artery haemoglobin (Hb) oxygen saturation (*S*a_O_2__; B) with increasing *T*_b_. (C) Change in *S*a_O_2__ with increasing *f*_H_ during arousal. Symbols represent mean and shading represents s.e.m., *n*=9 for each 1°C *T*_b_ increase. The red (A) and teal (B) lines represent mean summer *f*_H_ and *S*a_O_2__, respectively, *n*=5, with shading representing s.e.m. Solid black lines represent the relationships of *f*_H_ and *S*a_O_2__ with *T*_b_, with dashed lines representing the 95% confidence interval, non-linear regression.

During arousal from torpor to IBE and in the stable summer state, we measured *f*_H_ and *S*a_O_2__ using a MouseOx^®^ Plus pulse oximeter collar (Starr Life Sciences, Oakmont, PA, USA; Harvard Apparatus Canada, Saint-Laurent, QC, Canada). Pulse oximetry measures the *S*a_O_2__ of Hb molecules in red blood cells transcutaneously based on the principle that oxygenated and deoxygenated Hb absorb differing amounts of red and near-infrared (IR) light (Chan et al., 2013). The MouseOx^®^ collar used in this study was secured around the ground squirrel's neck, obtaining a strong signal from the carotid arteries, which supply blood to the head. A limitation of this methodology is the reliance on stable *f*_H_ for accurate measurements. The MouseOx^®^ is optimized to work at *f*_H_ between 90 and 900 beats min^−1^, but torpid 13-lined ground squirrels have *f*_H_ as low as 2–4 beats min^−1^ (Fig. 2A; MacCannell et al., 2018). As a result, the collar could not identify *f*_H_ or *S*a_O_2__ during torpor but began taking measurements when *f*_H_ reached approximately 100 beats min^−1^ early in arousal.

We removed torpid 13-lined ground squirrels from a walk-in environmental chamber (5°C) to an adjacent procedure room. Measurements of *S*a_O_2__ were made at an ambient temperature of 22°C, similar to the housing temperature of summer animals. Hair was removed from the neck using electric clippers and Nair^©^ hair remover for all animals. During arousal, *T*_b_ was measured continuously (once per second) throughout the experiment using a rectal probe, with data acquired using Expedata (Sable Systems, NV, USA). This procedure induced arousal in torpid animals. In summer 13-lined ground squirrels, *T*_b_ was measured at the beginning and end of the 40 min trials.

### Haematological analysis

Torpid and a separate group of anaesthetized (5% isoflurane, 95% O_2_) summer squirrels were killed with cervical dislocation, and blood was drawn using a cardiac puncture to the right ventricle. We measured Hb, Hct, Na^+^, K^+^, Ca^2+^ and glucose in venous whole blood using a portable blood analyser (i-STAT-1; Abbott Laboratories, Abbott Park, IL, USA) equipped with CG8+ cartridges. Although the i-STAT system can measure blood gases, the very low blood pressure and flow during torpor made it impractical to collect blood in a gas-tight fashion, and the samples visibly changed colour within the syringe during sampling, indicating a change in oxygenation. Therefore, we excluded the blood gas data.

### Data extraction and statistical analysis

*T*_b_ values were collected every second, and mean values for each 2.5 min interval were calculated. *f*_H_ and *S*a_O_2__ were collected every 0.2 s, with mean and s.e.m. values also calculated in 2.5 min intervals. The residuals of these data were normally distributed (Shapiro–Wilks test; Fig. S1A,B). To visualize the relationships among *T*_b_, *f*_H_ and *S*a_O_2__, *T*_b_ was grouped in 1°C bins and analysed using non-linear regressions (second-order polynomial models) with least square fitting. Linear regression was also used to assess the effect of *f*_H_ on *S*a_O_2__.

Haematological data were normally distributed (Shapiro–Wilks test; Fig. S1C–H). Unpaired, two-tailed *t*-tests compared torpor and summer heart masses and blood metrics (Hct, Hb, sodium, potassium, calcium and glucose). All data analysis and plots were generated using Graph Pad Prism (version 9.5.1).

## RESULTS AND DISCUSSION

### Arousals involve transient hypoxia–reoxygenation

The pulse oximeter did not provide reliable values until *f*_H_ reached approximately 100 beats min^−1^, at which point *T*_b_ had already increased from 4°C to 6°C. As a result, between 5 and 15 min elapsed between the time the animal was removed from the environmental chamber and when the first *f*_H_ and *S*a_O_2__ values were obtained. Despite this limitation, we found that *f*_H_ increased 3.4-fold over approximately 80 min, from 109±9 to 368±23 beats min^−1^ when *T*_b_ approached 37°C in IBE (Fig. 2A; Fig. S2A). When *T*_b_ exceeded 11°C, arousing *f*_H_ began to increase at rates similar to previous 13-lined ground squirrel measurements (MacCannell et al., 2018; Fig. 1B). The *f*_H_ of IBE 13-lined ground squirrels was similar to resting summer values (395±9 beats min^−1^; red line Fig. 2A). There was a positive, quadratic relationship (*R*^2^=0.95, d.f.=29, *P*=0.09) between *T*_b_ and *f*_H_ represented by Eqn 1:
(1)

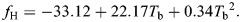
During arousal, *S*a_O_2__ changed rapidly. During the ∼80 min of arousal from torpor, carotid *S*a_O_2__ began at 44±7.6% when *T*_b_ was approximately 6°C, briefly declined to 33.9±1.4% when *T*_b_ was 8°C, and then increased 2.6-fold, to a maximum of 87±1.8% when *T*_b_ first reached 37°C (Fig. 2B; Fig. S2B). The mean summer *S*a_O_2__ (97±0.4%; Fig. 2B teal line, s.e.m. obscured by line representing mean) of 13-lined ground squirrels was constant over 40 min of measurement and was similar to values from an outbred lab mouse strain measured using the same MouseOx^®^ (95%; Ivy and Scott, 2017). However, *S*a_O_2__ of IBE 13-lined ground squirrels was 10% lower than summer *S*a_O_2__ (Fig. 2B). During arousal, there was a positive quadratic relationship between *T*_b_ and *S*a_O_2__ (*R*^2^=0.94, d.f.=28, *P*=0.03), represented by Eqn 2:
(2)

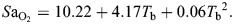
During arousal, *S*a_O_2__ increased linearly with *f*_H_ (*F*_1,30_=602.40, *P*<0.0001, *R*^2^=0.95; Fig. 2C) and was estimated by Eqn 3:
(3)

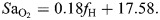
Fig. 2 suggests blood O_2_ flowing through the carotid arteries of 13-lined ground squirrels was low during torpor, but as *f*_H_ increased during arousal, *S*a_O_2__ rose proportionately. When *T*_b_ was 6°C, *S*a_O_2__ values were extremely low (45%). To our knowledge, this is the first report of O_2_ saturation in 13-lined ground squirrels, and this O_2_ saturation is lower than that of any other hibernator reported to date (Ma et al., 2005; Maginniss and Milsom, 1994; Webb and Milsom, 1994; Darden, 1972; Revsbech et al., 2013).

Beyond the low *S*a_O_2__ values early in arousal, *S*a_O_2__ declined further to 34% as *T*_b_ warmed to 10°C (Fig. 2B; Fig. S2B). This decline suggested that the early stages of arousal represented a phase where demand for O_2_, likely due to increased thermogenesis by brown adipose, outpaced O_2_ delivery. This decline in *S*a_O_2__ also coincided with a fairly constant *f*_H_ at the beginning of arousal (Fig. 2A; Fig. S2A). This delay in *f*_H_ increase likely results from depressed myocardial contraction and relaxation kinetics at low temperatures, even though 13-lined ground squirrel hearts exhibit robust function at low temperatures that render non-hibernator hearts highly arrhythmic (Caprette and Senturia, 1984; Fedorov et al., 2005). Interestingly, the early decline in *S*a_O_2__ during arousal found here aligns closely with another recent study in 13-lined ground squirrels, which found hyperventilation at the same *T*_b_ during arousal, suggesting decreased blood CO_2_ (Sprenger and Milsom, 2022). Additionally, the low *T*_b_ values experienced early during arousal would have challenged O_2_ offloading at the tissues, amplifying tissue hypoxia.

Throughout 80 min of arousal, *S*a_O_2__ increased by 260%, which suggested 13-lined ground squirrels experienced hypoxia–reoxygenation. In IBE, when breathing room air, 13-lined ground squirrel *S*a_O_2__ (87%) was similar to that of arctic ground squirrels (83%; Ma et al., 2005). In the wild, summer 13-lined ground squirrel burrow O_2_ levels are estimated to be 18.9% (Studier and Procter, 1971), compared with 20.9% in well-mixed air. To our knowledge, O_2_ levels in winter burrows have not been measured. However, snow cover could inhibit gaseous mixing in winter, so ambient O_2_ levels may be even lower during the hibernation season, further exacerbating hypoxia challenges. In other hypoxia-tolerant species, such low *S*a_O_2__ levels have only been reported in animals exposed to severely hypoxic environments. For example, in high-altitude deer mice, ∼65% *S*a_O_2__ was reached only when breathing 8% O_2_ (Ivy and Scott, 2021), and in naked mole rats, ∼40% *S*a_O_2__ was reached only when breathing 3% O_2_ (Pamenter et al., 2019).

### Haematological values differ slightly between torpor and summer

**Fig. 3.**
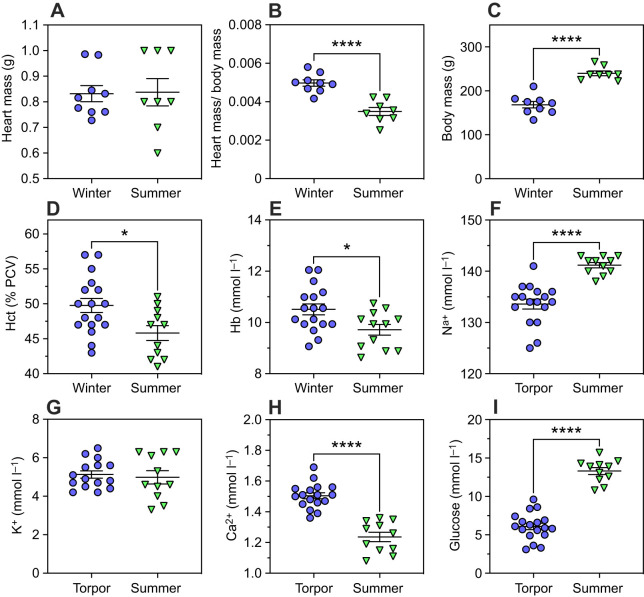
**Comparisons between torpid and summer haematological values.** (A) Heart mass, (B) heart mass relative to body mass, (C) body mass, (D) haematocrit (Hct, as percentage packed cell volume, PCV) and concentrations of (E) haemoglobin (Hb), (F) sodium, (G) potassium, (H) calcium and (I) glucose in venous whole blood. Means±s.e.m. Asterisks indicate a significant difference, *P<0.05, ****P<0.0001, two-tailed *t*-tests.

Heart mass was similar between torpor and summer 13-lined ground squirrels (*T*_15_=0.09, *P*=0.93; Fig. 3A). Body mass-corrected heart mass was 29.8% greater in torpor than in summer (*T*_15_=5.70, *P*<0.0001; Fig. 3B) due to the corresponding 29.8% lower body mass in torpor than in summer (*T*_15_=7.72, *P*<0.0001; Fig. 3C). Whole-blood Hct in torpor (49.76±1.01%) was 8.8% higher (*T*_26_=2.60, *P*=0.015; Fig. 3D) than summer values (45.82±1.07%), and Hb concentration showed similar trends (Fig. 3E).

Many experiments have previously characterized Hb concentration in hibernators with mixed results (reviewed in Milsom and Jackson, 2011). Our Hct results align closely with a recent study in 13-lined ground squirrels (Cooper et al., 2016a). The modest increase in torpor compared with summer is unlikely to affect blood flow significantly on its own, but Spurrier and Dawe (1973) noted that Hct tended to rise during arousal. This Hct increase, accompanied by low blood temperature early during arousal, could increase blood viscosity, further slowing rates of O_2_ delivery to tissues.

Whole blood collected during torpor had 6.7% lower Na^+^ concentrations than in summer (*T*_27_=5.94, *P*<0.0001; Fig. 3F). K^+^ concentration did not differ between groups (*T*_24_=0.39, *P*=0.27; Fig. 3G), but Ca^2+^ was 17.8% higher in torpor than in summer (*T*_27_=2.45, *P*=0.021; Fig. 3H). Finally, glucose concentrations from torpid blood were 54.3% lower than in summer blood (*T*_27_=11.26, *P*<0.0001; Fig. 3I).

It seems that the modest increase in Hct and Hb during torpor cannot be attributed to dehydration, as plasma Na^+^ levels were actually lower in this state, consistent with recent reports which indicate that 13-lined ground squirrel blood osmolarity declines during torpor despite the absence of water intake (Feng et al., 2019). We found that Ca^2+^ levels were higher in torpor than in summer, even without a dietary source. This finding supports the hypothesis that hibernators have developed mechanisms to maintain calcium homeostasis (Arfat et al., 2020). Blood glucose levels were significantly lower during torpor compared with those in summer (Fig. 3I), and these values align closely with previous measurements in 13-lined ground squirrel blood (Burlington and Klain, 1967; Nizielski et al., 1986). Low glucose levels may suggest a reliance on anaerobic metabolism. However, the evidence of lactate accumulation during torpor varies by species and blood sample type; for example, lactate accumulates in the red blood cells of arctic ground squirrels during torpor (Gehrke et al., 2019), while concentrations remained low in whole blood of golden-mantled ground squirrels (*Callospermophilus lateralis*; Maginniss and Milsom, 1994) and in serum of 13-lined ground squirrels (Feng et al., 2019). Instead, the blood glucose levels likely reflect that, during hibernation, 13-lined ground squirrels do not typically feed; the concentrations we report for torpid 13-lined ground squirrels resemble those found in fasted rats (Arnall et al., 1988). However, the values we report for summer 13-lined ground squirrels are similar to those of diabetic rats (Yang et al., 2020), which is expected because of the high body fat content in summer 13-lined ground squirrels (MacCannell et al., 2017).

### Possible mechanisms of I–R tolerance in hibernation

The resistance of hibernators to I–R has been attributed to many physiological adaptations that likely evolved as protection from O_2_ fluctuations during their torpor–IBE cycles, illustrated in Fig. 2B. Ground squirrel cardiac I–R tolerance is well defined in the literature (Bonis et al., 2019; Han et al., 2022; Xie et al., 2021; Salzman et al., 2017; reviewed in Drew et al., 2013).

Low glucose levels during torpor may indicate glycogenesis to supply energy during early arousal. One study found that by the third day of torpor, glycogen levels in 13-lined ground squirrel hearts were significantly higher than in summer, correlating with improved maintenance of cardiac function following *ex vivo* I–R in torpor (Heinis et al., 2015). Higher cardiac glycogen may help heart function during the early, hypoxic stages of arousal. Cardiac mitochondrial respiration is suppressed by 30% during torpor (Brown and Staples, 2014), which may help to conserve O_2_. ROS production during O_2_ fluctuations is also minimized in hibernator heart mitochondria by increased uncoupling proteins (Ballinger et al., 2017) to dissipate proton motive force and avoid reoxygenation-associated oxidative damage. There is also a hypothesis that repeated arousals themselves resemble ischaemic preconditioning, a condition in which previous bouts of tissue hypoxia prime tissues to resist later I–R insult (reviewed in Bhowmick and Drew, 2019). It is possible that repeated arousal throughout the hibernation season mimics preconditioning and mitigates oxidative stress by upregulating heat shock proteins and the HIF-1α pathway (reviewed in Jankovic et al., 2024).

## Supplementary Material

10.1242/jexbio.249830_sup1 Supplementary information
