# Supplementary material for: Arousal from hibernation increases blood oxygen saturation in 13-lined ground squirrels
Source: J Exp Biol. 2025 Apr 28;228(8):jeb249830. doi: 10.1242/jeb.249830 (PMC12079662; doi:10.1242/jeb.249830)
Supplement: Supplementary information [file jexbio-228-249830-s1.pdf]

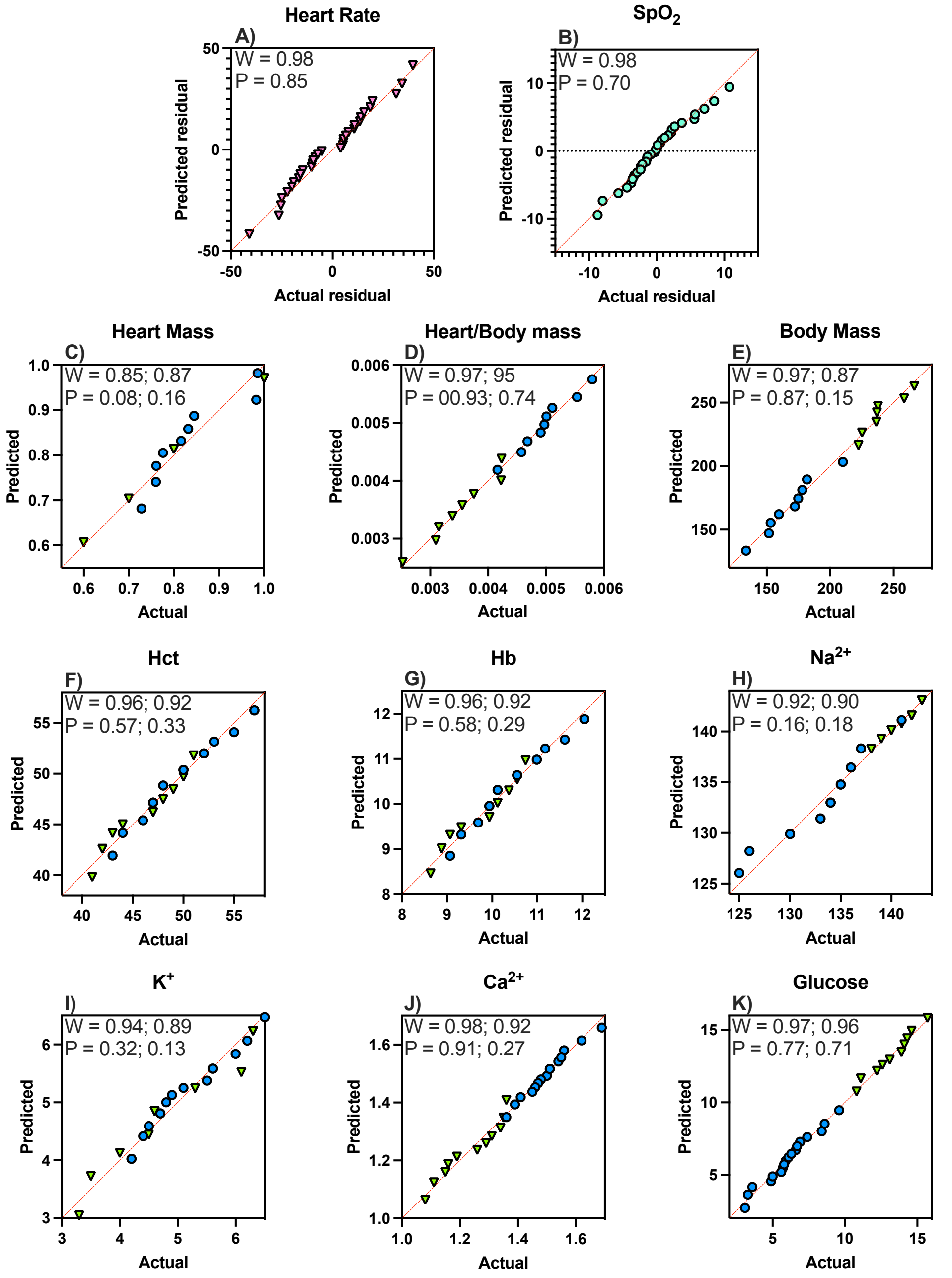

Fig. S1. Shapiro-Wilks normailty test results and qqplots for residuals (A,B), and raw data (C-K). W and P values are reported as torpor; summer.

Journal of Experimental Biology • Supplementary information

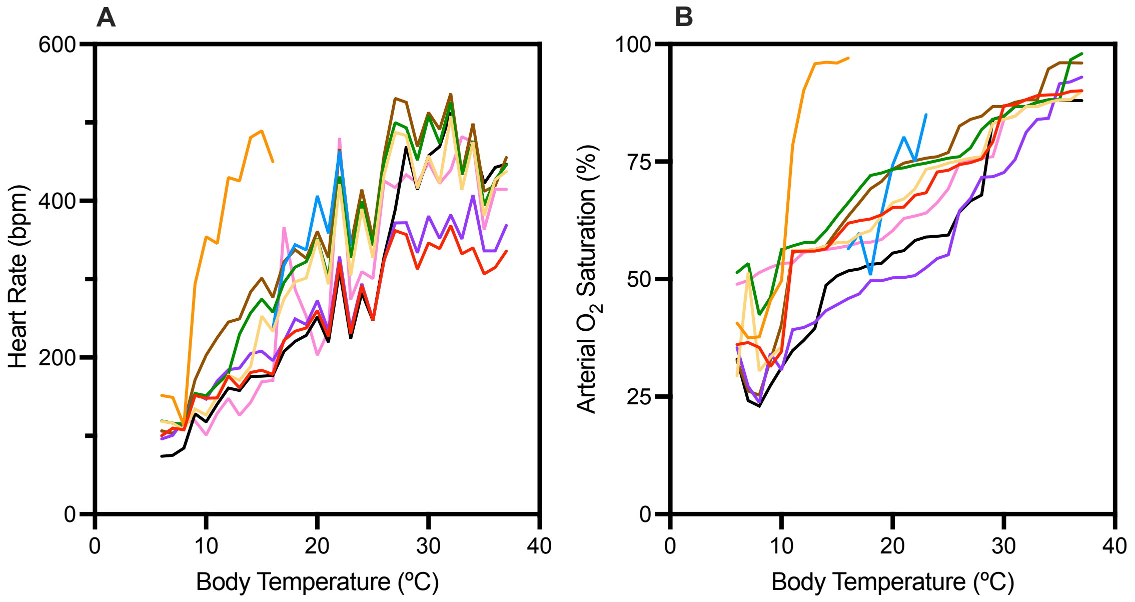

Fig. S2. Individual 13-lined ground squirrel mean values for each 1°C T_b increase during arousal. Heart rate (A) and carotid artery hemoglobin saturation (B), n = 9. Each different colour represents a different ground squirrel.
